# Work-Related Tuberculosis among Health Workers Employed in a Tertiary Hospital in Northeastern Thailand: A Report of Nine Cases

**DOI:** 10.3390/ijerph17145156

**Published:** 2020-07-17

**Authors:** Thanthun Sangphoo, Naesinee Chaiear, Patimaporn Chanpho

**Affiliations:** 1 Division of Occupational Medicine, Department of Community Medicine, Faculty of Medicine, Khon Kaen University, Khon Kaen 40002, Thailand; thanthun@kkumail.com; 2 Occupational Health and Safety Office, Faculty of Medicine, Khon Kaen University, Khon Kaen 40002, Thailand; patich@kku.ac.th

**Keywords:** tuberculosis, work-related TB, health workers, prolonged exposure, aerosol-generating procedure, respiratory protection

## Abstract

Between October 2016 and September 2018, fifteen health workers were diagnosed with tuberculosis (TB) at a tertiary hospital in northeastern Thailand. However, the cases could not be diagnosed as occupational TB according to international standards because of hospital limitations. The use of occupational epidemiological information provides a more effective work-related TB diagnosis. This study aims to provide a report of work-related TB using individual case investigation methods. We collected secondary data from the Occupational Health and Safety Office of the hospital in question, including baseline characteristics for the health workers, occupational history, source of TB infection and occupational exposure, and working environmental measurements. We found that nine of the fifteen cases were diagnosable as work-related TB due to two important factors: daily prolonged exposure time to an infected TB patient, and aerosol-generating procedures without adequate respiratory protection. The other six cases were not diagnosable as work-related TB because of inadequate evidence of activities related to the TB infection. The diagnosis of work-related TB thus requires occupational epidemiological information in order to complete the differentiation process.

## 1. Introduction

Tuberculosis (TB) is a major global health problem. The World Health Organization (WHO) has determined that, with 87 percent of its population exposed, Thailand is one of twenty-two countries with a TB burden. The Thai Tuberculosis Division of the Thai Ministry of Public Health’s Department of Disease Control, reported 120,000 TB patients in 2016 [1], and the number trends to increase each year.

Health workers are often at risk of exposure to the infectious phase of TB during the treatment process for TB patients, leading to work-related TB infections [2,3,4]. A study about TB infections in health workers found that the risk for this group is two to four times higher than for the general population [5,6,7]. A health surveillance program for health workers is therefore essential for preventing the contraction of the disease from TB patients during work activities and in the workplace in general [8,9]. In some countries, there are guidelines to control the spread of TB among health workers by Bacillus Calmette–Guerin (BCG) vaccination, but it is not well implementation. In Thailand, BCG vaccination is prescribed as the primary vaccine for newborns mainly to prevent TB meningitis. There is no BCG vaccination policy for adults, including health workers [10]. Aside from BCG vaccination, the respiratory protection program (RPP) as mandated in the U.S. is not mandated for health workers in Thailand [11,12,13] except in the step of seal-check, for which the training is compulsory. In addition, some health workers are accidentally exposed to TB patients while using only a surgical mask, leading substantially to TB infection among health workers. Therefore, all health workers must be provided with appropriate respirators including filtering facepiece respirators (i.e., N95 respirator) together with RPP enforcement [14,15,16,17,18].

The Centers for Disease Control and Prevention (CDC) in the U.S. set guidelines to test all health workers with a history of TB exposure in the workplace. In Thailand, the Tuberculosis Division of the Department of Disease Control set similar guidelines [19], using chest X-ray and physical examinations for an annual examination program for health workers with accidental exposure to TB or “close-contact” at work [20,21]. Another guideline from the Department of Medical Services [22] uses an annual chest X-ray for all health workers and every six months for health workers exposed to TB. In addition, one of the tertiary hospitals developed guidelines for screening latent TB infection (LTBI) as part of a preplacement medical examination and as part of medical surveillance after TB exposure [23,24,25]. However, TB continues to be found occasionally among health workers in Thailand as the spread of TB infections in the work environment persists [26,27].

Fifteen health workers at a tertiary hospital in northeastern Thailand from various departments were diagnosed with pulmonary and/or extrapulmonary TB between October 2016 and September 2018. The individual case investigations from the Occupational Health and Safety Office of the hospital cited a history of TB exposure for the fifteen cases, through contact during work and/or at home. The ambiguity of the information puts into question whether the disease was due to occupational exposure. The international guidelines for diagnosing occupational TB were developed by the CDC [28]; therefore, pre-placement (baseline) and subsequent periodic testing for TB is required for all health workers. Testing should also be performed after exposure to a patient known to be infected. The resulting information can be used to confirm/refute whether the TB diagnosis is the result of occupational exposure.

The United States Occupational Health and Safety Administration (U.S. OSHA) [5] confirms diagnoses of occupational TB using DNA fingerprinting. The test is a comparison between the DNA sequence of TB from the health worker and the infected TB patient with whom the health worker came into contact. Unfortunately, the particular hospital in northeastern Thailand does not have access to such testing due to cost limitations, so a definitive diagnosis of occupational TB according to international guidelines cannot be extended to include the TB prevention policy at the hospital. Nevertheless, individual case investigations according to TB epidemiology could be carried out instead [29,30], which include information about the host, exposure agent, and the occupational environment extending to all related work procedures [31,32,33,34]. This information and investigative data could be combined to support a diagnosis of work-related TB, which is valuable as medical evidence for the preparation of health surveillance programs to control the spread of TB among health workers [35]. The objective of our study was to provide an individual case investigation report for each of the fifteen cases of TB so as to determine which were indeed work-related TB based on their respective occupational epidemiology.

## 2. Materials and Methods

We investigated fifteen cases of suspected workplace TB following the individual case investigation method. The data reviewed were (a) the secondary data from the Occupational Health and Safety Office of the hospital and (b) the patients’ medical records.

### 2.1. Inclusion

We included health workers from the hospital diagnosed with TB between October 2016 and September 2018.

### 2.2. Study Tools and Data Collection

An identification code was assigned to the data collected for each of the fifteen TB cases. The data were divided into two sets. The first set included the baseline characteristics of the fifteen TB cases: sex, age, underlying disease, history of previous TB infections, history of household contact to TB, diagnosis, and related laboratory investigations. Diagnostic investigations for pulmonary TB [36] included sputum acid-fast bacillus tests (AFB), sputum culture for TB (C/S), polymerase chain reaction tests for TB (PCR), chest X-ray (CXR), and other related investigations. The second set included individual case investigation data [37]: 1. Occupational history—job title at the time that TB was diagnosed, duration of employment (years), and duration of exposure (years) [9,38]. 2. Source of TB and occupational exposure—average exposure times per day (hours) [9,38], number of infected TB patients to whom the worker was exposed three months prior to them being diagnosed with the disease [39,40], compliance in properly wearing respiratory protection (i.e., using filtering facepiece respirators, and seal-check) to protect the upper respiratory tract from infection, and aerosol-generating procedures used on TB patients [41,42] (i.e., sputum suction, bronchoscope, endotracheal tube intubation, aerosolized medication, procedure with infectious sputum, and autopsy). 3. Working environment: the separating arrangements or isolation room for TB or respiratory infection patients [43], and ASHRAE 2013 indoor air quality (IAQ) results (i.e., airborne particle count (count/m^3^), room temperature (°C), room relative humidity (%RH), room pressurization: negative (Pa), and air changes per hour (ACH)).

### 2.3. Analysis

Data were recorded using Microsoft Excel version 2016 (licensed to Khon Kaen University, Khon Kaen, Thailand). Descriptive statistics, including frequency and average, were used to assess the data.

### 2.4. Ethics Considerations

The authors considered confidentiality as a priority. This report was certified by Institutional Review Board Number; IRB00001189 and Khon Kaen University Research Committees under the reference of HE621543.

## 3. Results 

Fifteen TB cases were considered in the current study: three males and twelve females. The average age was 38.7 years (SD 11.02; range 24–59 years) (Table 1).

Thirteen cases were diagnosed with only pulmonary TB, one with only pleural TB, and one with pulmonary TB combined with lymph node TB. Nine cases were diagnosed with work-related TB, while six cases were not. These six cases were not considered to be occupational because (a) one had previously been diagnosed with TB, (b) three had a definite history of household TB exposure, and (c) two were found to be laboratory-negative despite a clinical diagnosis.

Individual case investigation results showed that only nine had work-related TB. Job titles included four practical nurses, two registered nurses, two technologists (medical and radiological), and one medical resident (Table 2). The duration of employment ranged between 2 and 21 years (average 8.2; SD 6.1 years). The duration of exposure ranged between 2 and 19 years (average 6.6; SD 5.29 years). All of the health workers were exposed to infected TB patients while working 4–10 h per day (average 6.4; SD 1.94 h per day).

Aside from work duration, we found that during the last three months prior to infection, each of the workers had been exposed to one or more infected TB patients. We also found that all health workers with work-related TB performed at least one aerosol-generating procedure with misuse of respiratory protection, such as using a surgical mask instead of filtering facepiece respirators.

We found that only five of the nine workplaces had a designated isolation room for patients with TB or other respiratory infections. The indoor air quality (IAQ) measurements as per the ASHRAE 2013 guidelines showed that all parameters were being measured in only five of the nine workplaces, and only two of these met the requirements of the guidelines.

## 4. Discussion

The CDC occupational TB diagnosis guidelines require baseline and periodic testing for TB, followed up with testing after health workers are exposed to infected TB patients. The U.S. OSHA guidelines require DNA fingerprinting in order to confirm whether a TB diagnosis is related to patient exposure at work. These two guidelines clearly link the diagnosis of occupational TB with evidence of it. Thailand does not, however, have access to either test, so the diagnosis of occupational TB is problematic in light of insufficient medical evidence. The use of an individual case investigation—according to the TB epidemiology—can be used to diagnose work-related TB [29,37]. Based on such an investigation, nine out of fifteen health workers were diagnosed with work-related TB. Considering the duration of work as well as both the duration of employment and duration of exposure, the period was too broad, so it was not possible to determine whether these durations were related to the likelihood of TB infection. However, when considering average exposure time per day, we found that all nine workers with occupational TB were exposed to infected TB patients for four or more hours per day, which is consistent with the 2005 CDC screening guideline for TB [28]. The guidelines specify that prolonged daily time exposed to infected TB patients is one of the factors that increases the risk of TB infection in health workers. All nine cases are thus considered to be due to prolonged exposure, which is the foremost cause in the diagnosis of work-related TB.

The current study found that all nine health workers with work-related TB were performing at least one aerosol-generating procedure while not complying with respiratory protection protocols. The protocols were enforced on health workers to prevent air-borne infection from work, which must be defined as the hospital policy for provide filtering facepiece respirators and seal-check [17,18]. However, the regulations about Occupational Health and Safety in Thailand are not mandating completely respiratory protection protocols, so these are not supported. Panthong et al. (2019) [44] conducted a study on the prioritization of the risk of TB exposure in hospitals and found that procedures that generate aerosols accelerate the spread of TB from infected patients. Such work is thus considered to involve a high risk of TB exposure; moreover, the poorer the adherence to protective measures the greater the risk [27]. Health workers who perform aerosol-generating procedures without consideration of respiratory protection [9,39] constitute a further important factor in the diagnosis of work-related TB. With respect to the working environment, we had insufficient information as IAQ measurements had not been performed at all workplaces where health workers were found to have work-related TB.

Although achieving an occupational TB diagnosis using an international standard would be ideal [43,45], a work-related TB diagnosis represents a practicable alternative if the hospital has limited laboratory resources. Such a diagnosis requires occupational epidemiological information including occupational history, source of TB infection, occupational exposure, and details on the working environment [8,35].

A limitation of the study is the use of historical, secondary data from the Occupational Health and Safety Office of the respective hospital and its medical records. Some of the information was incomplete, either not entered at the time or not measured.

## 5. Conclusions

These case reports were conducted in a tertiary hospital in northeastern Thailand between October 2016 and September 2018. Individual case investigations confirmed that nine out of fifteen health workers had actual work-related TB according to their occupational epidemiology. There are two important issues concerning the diagnosis of work-related TB: daily prolonged exposure time to infected TB patients, and aerosol-generating procedures, particularly when respiratory protection protocols are not being followed. There was insufficient information on working environment issues, so further research is needed before generalizations can be made.

## Figures and Tables

**Table 1:** Baseline characteristics

No.	Sex	Age	Underlying Disease	Previous TB Infections	History of Household Contact to TB	Confirmed Investigation	Diagnosis	Work-Related TB
1.	Female	56	No	No	No	+AFB, +CXR	TB lung	Yes
2.	Female	39	No	No	No	+AFB, +CXR	TB lung	Yes
3.	Female	28	No	No	No	-	TB lung	No
4.	Female	24	No	No	No	+AFB, +C/S, +PCR, +CXR	TB lung	Yes
5.	Female	59	Breast cancer	Yes	No	+AFB, +C/S, +PCR, +CXR	TB lung	No
6.	Male	35	No	No	No	+C/S, +PCR	TB lung	Yes
7.	Male	32	No	No	Yes	+Pleural fluid culture for TB	TB pleura	No
8.	Female	27	No	No	No	+C/S, +Lymph node FNA	TB lung, lymph node	Yes
9.	Female	34	Thalassemia	No	No	+PCR, +CXR	TB lung	Yes
10.	Female	56	No	No	No	-	TB lung	No
11.	Female	38	No	No	No	+C/S, +PCR	TB lung	Yes
12.	Female	37	No	No	Yes	+CXR	TB lung	No
13.	Female	41	No	No	No	+AFB, +CXR	TB lung	Yes
14.	Male	29	No	No	No	+AFB, +C/S, +PCR, +CXR	TB lung	Yes
15.	Female	45	No	No	Yes	+CXR	TB lung	No

**Table 2:** Individual case investigation for work-related TB.

No.	Job Title	Exposure				Working Environment	
		Duration of Employment (Years)	Duration of Exposure (Years)	Average Exposure Time Per Day (hours)	Number of Index Case Exposure within 3 Months	Isolation Room for TB Patient	IAQ (ASHRAE 2013)
1.	Assistant nurse	4	4	6	2	Yes	Failed
2.	Assistant nurse	5	5	6	3	No	Not evaluated
4.	Registered nurse	2	2	4	12	Yes	Failed
6.	Medical technologist	11	11	8	36	No	Failed
8.	Registered nurse	3	3	6	5	Yes	Not evaluated
9.	Radiologic technologist	10	5	4	1	No	Passed
11.	Assistant nurse	13	5	6	2	Yes	Not evaluated
13.	Assistant nurse	21	19	8	3	Yes	Passed
14.	Medical resident	5	5	10	15	No	Not evaluated
